# Cancer in Africa: The Untold Story

**DOI:** 10.3389/fonc.2021.650117

**Published:** 2021-04-15

**Authors:** Yosr Hamdi, Ines Abdeljaoued-Tej, Afzal Ali Zatchi, Sonia Abdelhak, Samir Boubaker, Joel S. Brown, Alia Benkahla

**Affiliations:** ^1^ Laboratory of Biomedical Genomics and Oncogenetics, Institut Pasteur de Tunis, University of Tunis El Manar, Tunis, Tunisia; ^2^ Laboratory of Human and Experimental Pathology, Institut Pasteur de Tunis, Tunis, Tunisia; ^3^ Laboratory of BioInformatics bioMathematics, and bioStatistics (BIMS), Institut Pasteur de Tunis, University of Tunis El Manar, Tunis, Tunisia; ^4^ Engineering School of Statistics and Information Analysis, University of Carthage, Ariana, Tunisia; ^5^ Department of Integrated Mathematical Oncology, Moffitt Cancer Center, Tampa, FL, United States

**Keywords:** cancer, Africa, epidemiology, incidence rates, mortality rates, risk factors, medical devices, human development index

## Abstract

**Background:**

Despite rising incidence and mortality rates in Africa, cancer has been given low priority in the research field and in healthcare services. Indeed, 57% of all new cancer cases around the world occur in low income countries exacerbated by lack of awareness, lack of preventive strategies, and increased life expectancies. Despite recent efforts devoted to cancer epidemiology, statistics on cancer rates in Africa are often dispersed across different registries. In this study our goal included identifying the most promising prevention and treatment approaches available in Africa. To do this, we collated and analyzed the incidence and fatality rates for the 10 most common and fatal cancers in 56 African countries grouped into 5 different regions (North, West, East, Central and South) over 16-years (2002–2018). We examined temporal and regional trends by investigating the most important risk factors associated to each cancer type. Data were analyzed by cancer type, African region, gender, measures of socioeconomic status and the availability of medical devices.

**Results:**

We observed that Northern and Southern Africa were most similar in their cancer incidences and fatality rates compared to other African regions. The most prevalent cancers are breast, bladder and liver cancers in Northern Africa; prostate, lung and colorectal cancers in Southern Africa; and esophageal and cervical cancer in East Africa. In Southern Africa, fatality rates from prostate cancer and cervical cancer have increased. In addition, these three cancers are less fatal in Northern and Southern Africa compared to other regions, which correlates with the Human Development Index and the availability of medical devices. With the exception of thyroid cancer, all other cancers have higher incidences in males than females.

**Conclusion:**

Our results show that the African continent suffers from a shortage of medical equipment, research resources and epidemiological expertise. While recognizing that risk factors are interconnected, we focused on risk factors more or less specific to each cancer type. This helps identify specific preventive and therapeutic options in Africa. We see a need for implementing more accurate preventive strategies to tackle this disease as many cases are likely preventable. Opportunities exist for vaccination programs for cervical and liver cancer, genetic testing and use of new targeted therapies for breast and prostate cancer, and positive changes in lifestyle for lung, colorectal and bladder cancers. Such recommendations should be tailored for the different African regions depending on their disease profiles and specific needs.

## Introduction

Cancer is an emerging health problem in Africa that needs to be addressed appropriately in order to control for increased incidence and mortality rates (1, 2). It has been suggested that by 2030 there will be a 70% increase in new cancer cases due to population growth and aging (3). In Africa, this ever present disease has coexisted with more recently discovered communicable diseases such as Malaria, Ebola, AIDS and COVID19 (4, 5). Even though cancer death rates have surpassed those of AIDS, tuberculosis, and malaria combined, there remains a lack of commitment to fighting cancer in Africa. Indeed, most attention goes to investigating communicable diseases while disregarding the challenges posed by several non-communicable diseases such as cancer (6). Additionally, due to the cost of care and the absence of facilities, cancer mortality rates are expanding in Africa (7). Cancer death rates in Africa are projected to exceed the global average by 30% in the next 20 years (8). Cancer is a genetically driven disease that interacts with other risk factors to determine an individual’s risk.

Three of these associated risk factors speak to the need for making cancer detection and therapy a priority for African nations. The first concerns health care improvements. Based on data from the world bank, life expectancy of Africans has been growing faster than the global average, and is now thought to be about 60 years continent wide. For example, advancements in AIDS therapy and other factors have raised life expectancy for rural Kwa-Zulu Natal from 49 years in 2003 to 60.5 in 2011 (9). As cancer incidences and cancer mortality increase with age, such progress in life expectancy directly leads to more cancer cases. The second follows from the growth in wealth and prosperity in Africa. Changes in lifestyles are associated with increased cancer risks and exposures to carcinogens and mutagens. Such changes include increased urbanization, emergence of different sources of pollution exposure, increase and changes in tobacco and alcohol usage, and changes in diets towards more meat, sugar and processed foods. Environment and lifestyle associated cancer risks can both increase incidences in younger age classes and exacerbate cancer incidence in the elderly. Third, Africa includes diverse ethnicities and sub-populations manifesting a number of genetically associated cancers that disproportionately affect different groups over others. As other health risks decline, these group-dependent cancer rates will become more apparent and take a relatively larger toll on life.

The Global Initiative for Cancer Registry Development (gicr.iarc.fr), led by International Agency for Research in Cancer (IARC), is a partnership of leading cancer prevention organizations that seeks to address data availability, ensuring the robustness of cancer incidence data by improving their quality, comparability and use. Data collected in this framework is available through IARC’s GLOBOCAN database. The estimated number of cancer cases and deaths from the year 2002 through the year 2018 are available at the Global Cancer Observatory (http://gco.iarc.fr). In assembling regional and global profiles, the GLOBOCAN methods for incidence and mortality estimation rely upon the best available data from a given country (10, 11).

Records from 56 different African countries are available on GLOBOCAN. Cancer incidences in population-based cancer registries are mainly determined by the cancer cases reported from hospitals (population-based cancer registries: PBCR). Mortality statistics are collected and made available by the WHO. Here, our objective is to study the trends in cancer incidence and fatality rates in Africa. We collated data on 10 different cancer types from 56 African countries grouped into 5 different regions. From these data, we estimated cancer incidence (number of afflicted individuals per 100,000 at a given time point), and fatality rates (number of deaths from the cancer per year per number of afflicted individuals) over a span of 16 years (2002-2018). For many cancers, we can track incidence by gender. We use our statistical analyses of incidences, fatality rates, temporal trends and regional trends to prioritize regional and cancer-specific needs for treatment and prevention strategies. Additionally, we analyze the availability of medical devices used in cancer care across Africa’s regions; and we assess the association between the Human Development Index (HDI) and cancer incidence and fatality rates in Africa.

## Material and Methods

### Data Sources and Population

We extracted data from the 4 latest GLOBOCAN reports Global Cancer Statistics https://gco.iarc.fr) for 56 African countries covering cancer incidence and fatality rates for the last 16 years (2002-2018). The cancer incidence refers to the number of diagnosed cases per 100,000 inhabitants at that time point. The fatality rate is calculated from the ratio of deaths per year from the cancer divided by the number of persons afflicted with the cancer that year (deaths per year divided by the number of currently diagnosed cases). Given that one has the cancer, the fatality rate represents the probability of dying from that cancer per year. When multiplied by 100, the fatality rate represents a percentage of those with a particular cancer who die per year. We also examined temporal trends (2002-2018) for 10 cancer types by selecting registries with long standing and high quality data over the period.

### Statistical Analyses

From collected GLOBOCAN data, we created a database of incidence and mortality for each of the 10 cancers. We calculated cancer incidences (IR) and fatality rates (FR) by using the estimated population size by country, by African regions, and by year (12). For example, cancer incidence was measured by dividing the total number of people affected by a specific cancer by the total population and multiplying by 100,000. For the fatality rate we divided the total number of deaths from that cancer (by year) by the total number of individuals afflicted by the cancer during that year.

**Table S1** lists the countries by region. Data were structured according to the Northern, Western, Eastern, Central and Southern African regions. Cancer mortality data were available for just three cancer types: breast, prostate, and cervical cancers. The estimated incidence and fatality rates for 2002, 2008, 2012 and 2018 are presented using maps of Africa. Patterns in the recorded incidences by cancer type and sex are presented as bar charts. All analyses were performed using Python programming language (13) and R statistical language (14). For generating maps, we used the GeoPandas package in Python (15, 16).

We favored regional analyses over individual countries for three reasons: 1) aggregating data across a number of countries increases sample sizes and the calculation of regional averages reduces the fluctuations due to the quality of country by country reports, 2) countries within a region do share ethnic, socio-economic, and cultural affinities, and 3) any region by region differences likely represent strong signals of region-specific cancers and their temporal trends. That said countries within a region can show striking differences in socio-economic measures.

### Graphics and Basic Statistics

For each region, we summed cancer incidence for the countries in the area using Readerscan to assess the number of cases during the last 16 years. The increase or decrease of incidence rates is represented on the maps by the shade and contrast of the color. Similarly, average region-specific fatality rates for breast, prostate and cervical cancers were computed from the average of fatality rates among the countries of a specific region. The figures show where in Africa specific cancer types are most or least frequent suggesting where increased attention to treatment and prevention would be most effective.

### Available Medical Devices Data

The initial objective with gathering data on the availability of cancer medical device was to show that the higher mortality rates in some areas is due to a lack of equipment. Data on medical devices including equipment for Computed Tomography, Magnetic Resonance Imaging, Positron Emission Tomography, Gamma Camera or Nuclear Medicine, Linear accelerator, Telecobalt unit, Radiotherapy, Mammographs^1^ were extracted from the WHO (https://apps.who.int/gho/data/node.country). Data are available in **Table S14** (Central African region), **Table S15** (Eastern African region), **Table S16** (Northern African region), **S17** (Southern African region) and **Table S18** (Western African region). Statistics on this equipment are spotty. Such equipment is often required for the detection of certain cancers or necessary for care. At best there is some availability, and at worst the equipment is completely absent from the medical infrastructure in Africa.

### Human Development Index

The Human Development Index (HDI) is a summary measure of achievement in key dimensions of human development: a long and healthy life, standard of living, and education levels. The HDI is the geometric mean of normalized indices for each of these three dimensions. It also offers other composite indices as broader proxies for some of the key issues of human development such as wealth or income inequality, gender disparity, and poverty rates. Country specific HDI data were downloaded from UNESCO (http://uis.unesco.org/), see **Table S12**. In order to harmonize with our data, we calculated a regional HDI average for 2018 only (there was no HDI data for 2002, 2008 and 2012). We tested for associations between the HDI and the incidence rate, IR (**Figure 12A**). Using the least squares approach, **Figure 12B** shows the best fit relationship between fatality rates, FR, and HDI.

## Results

Cancers listed in this report are ordered first by those for which we have mortality data and then roughly in descending order of overall incidence. Data on cancer classification and ranking worldwide as well as the number of new cases and deaths have been cited based on the last GLOBOCAN report (https://gco.iarc.fr/today/home).

### Breast Cancer

During the last decades, breast cancer has become the most common type of cancer among women worldwide (18). It is a multifaceted disease involving environmental, genetic, and lifestyle risk factors. Breast cancer also represents a collection of clinically heterogeneous diseases ranging from indolent to aggressive. Several differences have been observed in breast cancer epidemiology between populations (19). It has been shown that American women of African origins are three times more likely than Caucasian Americans to develop highly aggressive triple-negative and inflammatory forms of breast cancer (20). Moreover, several studies have shown that high rates and long histories of consanguinity, observed in some upper income countries in Asia and elsewhere, decrease incidences of breast cancer by decreasing the frequency of mutations on the two major susceptibility genes *BRCA1* and *BRCA2* (21, 22).

#### Breast Cancer Incidence Rates (IR)

**Figure 1:**
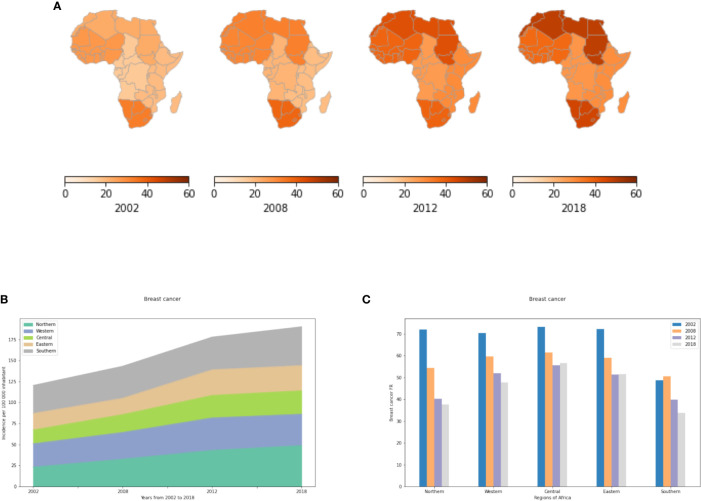
Incidence and fatality rates of breast cancer per 100,000 inhabitants from 2002 to 2018. IR refers to active cancer cases per year and per region per 100 000 inhabitants. The FR of a region per year is obtained by dividing the number of cancer deaths by the total number of active cancer cases per year in a specific region and multiplying by a hundred to give a percent. **(A)** Breast cancer incidence rates in the different African regions per 100,000. **(B)** Changes in incidence rate from 2002 to 2018. **(C)** Breast cancer fatality rates (percent mortality per year of those afflicted) in the different African regions given as a percent.

The incidence of breast cancer has increased dramatically in Northern and Southern Africa (**Figures 1A, B**). Incidence in North Africa has doubled from 2002 to 2018 with 23.3 cases per 100,000 inhabitants in 2002 to 48.9 cases per 100,000 inhabitants in 2018 (**Table S2**). This is mainly explained by the adoption of a western lifestyle in both Northern and Southern Africa such as nulliparity, breastfeeding, use of oral contraceptives, hormone replacement therapy (HRT) after menopause, nutrition, stressful lifestyle and pollution (23, 24). In Eastern, Central and Western Africa, the incidence has remained stable since 2002. Perhaps this can be explained by fewer changes in lifestyle and habits that increase incidences of breast cancer. These regions of Africa may have yet to see increases in obesity or decreases in physical activity (23). These regions may have a smaller proportion of urban dwellers and hence less exposure to urban pollution, mutagens and carcinogens.

#### Breast Cancer Fatality Rates (FR)

In all African regions, breast cancer fatality rates have decreased from 2002-2008, and then have remained relatively constant from 2012-2018 (**Figure 1C**). This observation demonstrates the importance of dedicating more efforts, such as early detection, to reducing mortality from this cancer. Northern and Southern Africa exhibit lower fatality rates than other African regions because of available facilities in terms of screening, diagnosis and treatment (including imaging, disease-specific pathologists, surgery, chemotherapy, hormonal therapy and radiotherapy) as compared to Eastern, Central and Western Africa. Finally, fatality rates may remain high across all of Africa due to the paucity of facilities related to precision oncology such as genetic testing, targeted therapies, and immunotherapy.

### Prostate Cancer

Prostate cancer is a common malignancy among men and perhaps the third most aggressive neoplasm worldwide, causing approximately 90,000 deaths per year in Europe. International guidelines became more conservative over the past decades in the management of prostate cancer cases. Prostatectomy and/or external beam radiotherapy are the most common intervention, followed by maintenance on androgen deprivation therapy (ADT) known as chemical castration. Standard of care in prostate cancer includes a combination of next generation endocrine therapies like enzalutamide, with cytotoxic agent docetaxel. Medical and biological advances have led to new promising treatments for this cancer that include Radium-223 for bone metastases, pembrolizumab as immunotherapy (PDL1 blocker) for microsatellite instability (MSI) disease, and poly ADP ribose polymerase (PARP) inhibitors for those with mutations in homologous recombination genes, most commonly *BRCA2*.

Other than age, few risk factors have been characterized. The best known include smoking (25, 26), diet (27), obesity (28) and genetic predispositions. The most common mutations involved in prostate cancer include *BRCA1/2*; *ATM* (odds ratio (OR) = 2.18), *HoxB13* (OR = 3.23), genes involved in repairing mismatched genes and genes associated with Lynch Syndrome (OR = 4.87), and *CHEK2* (OR = 1.98) (29). Prostate cancer seems to have a strong ethnic association. Men of African ancestry are at an increased risk of the disease. In the US, African Americans are more likely to be diagnosed with prostate cancer and 2.5 times more likely to die from the disease. A recent literature review showed that African American men were less likely than European American men to seek treatment as a direct or indirect consequence of health disparities such as financial barriers, lack of health insurance, and/or poor health-seeking behavior (30). Furthermore, some men may be reluctant to seek treatment because of concerns regarding the side-effects of therapy such as incontinence and sexual dysfunction.

#### Prostate Cancer IR

**Figure 2:**
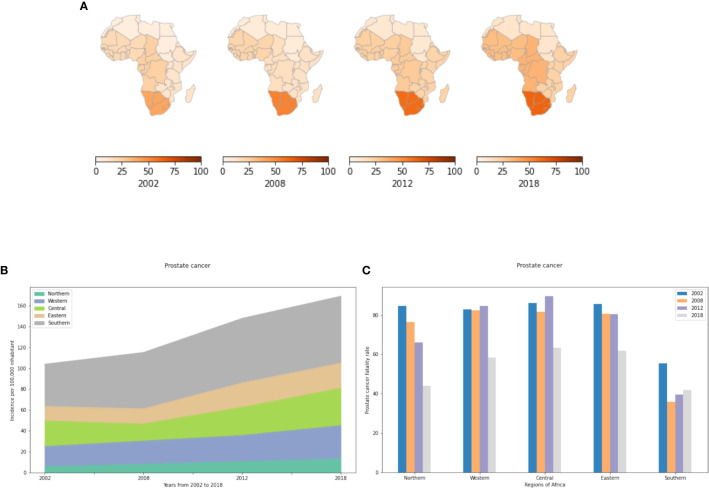
Incidence and fatality rate for prostate cancer per 100,000 inhabitant from 2002 to 2018 in Africa. **(A)** Prostate cancer incidence rates in the different African regions per 100,000 inhabitant. **(B)** Incidence rates from 2002 to 2018. **(C)** Prostate cancer fatality rates (percent mortality per year of those afflicted) in the different African regions.

**Figures 2A, B** show a low overall IR for prostate cancer in Northern Africa that has slowly increased from 5 to 13 cases per 100,000 inhabitants from 2002 to 2018 (**Table S3**). In Eastern, Central and Western Africa, prostate cancer is 2 to 6 fold more prevalent with an IR that reaches 35 cases per 100,000 population in 2018 in Central Africa. In Southern Africa, prostate cancer IR is alarming with a prevalence that is 5 times more than that of Northern Africa in 2018. The increased prostate cancer risk in Sub Saharan Africa may be explained by genetics, though the potential carcinogenic impact of environmental and lifestyle factors cannot be ignored. Indeed, it is well documented that the population with the highest reported incidence and mortality rates globally are African Americans. In 2009, Odedina and collaborators, suggested that the roots of the high burden of prostate cancer among African American can be explained (at least in part) by increased genetic susceptibility dating back to the approximately 360,000 transatlantic slaves, mainly from West/Central West Africa (31). In addition, the VhaVenda Vhembe District of the Limpopo Province in South Africa has practiced residential dichlorodiphenyltrichloroethane (DDT) spraying for malaria control since 1945 (32). The identification of a link between maternal DDT exposure and urogenital birth defects in newborn VhaVenda boys provides one of several links between pesticide use in Sub Saharan Africa and prostate cancer (33). In addition, a case-control study from Southern Africa showed that prostate cancer is associated with high intake of fat, meat, and eggs; eating out of the house; and low consumption of vegetables (34).

#### Prostate Cancer FR

In the literature, prostate cancer is the most deadly cancer for men in Southern Africa (12). It is also the most commonly diagnosed cancer and the leading cause of cancer death in men in Central Africa. It ranks before lung cancer in terms of fatality rates for men in Northern Africa. Our study shows that prostate cancer IR has been increasing steadily since 2002. Most cases are recorded from Southern Africa where the number of new cases has increased by more than 60% from 2002 to 2018. Unlike IR, the FR in Southern Africa is significantly less than in Northern, Western, Central and Eastern Africa (**Figure 2C**). In 2002, this cancer had over 80% FR in the five regions. While the FR is lower by 2018, more than 6 out of 10 cases died within 12 months after diagnosis in Central, Western and Eastern Africa and more than 4 out of 10 in the North and South.

### Cervical Cancer

Cervical cancer is the fourth most common cancer in women worldwide. Around 85% of the global burden occurs in low and middle income regions, where it accounts for almost 12% of all female cancers. In comparison, in upper income regions, cervical cancer accounts for less than 1% of all cancers in women (35). Cervical cancer, the only cancer that is almost entirely preventable and curable if detected early, affects mainly middle-aged women (30 to 50 years) (36). It is caused by sexually acquired infections from certain types of Human papillomaviruses (HPV) (37). Two HPV types, 16 and 18, are responsible for approximately 70% of cervical cancer cases and pre-cancerous cervical lesions, globally. There is also evidence linking HPV to other cancer types such as anus, vulva, vagina, penis and oropharynx cancers. Three HPV vaccines are now available in many countries throughout the world - a bivalent, a quadrivalent, and a nonvalent vaccine. All three vaccines are highly effective in preventing infection with HPV types 16 and 18. The vaccines are also highly efficient in preventing precancerous cervical lesions caused by these virus types. The WHO national immunization program against HPV includes most Eastern and Southern African countries. Libya is the only North African country using this vaccine to prevent cervical cancer (**Figure S1**). Ivory Coast, Gambia and Senegal are the only three Western African countries that have been included in this program. However, no vaccination against HPV has been recorded in Central Africa.

#### Cervical Cancer (IR)

**Figure 3:**
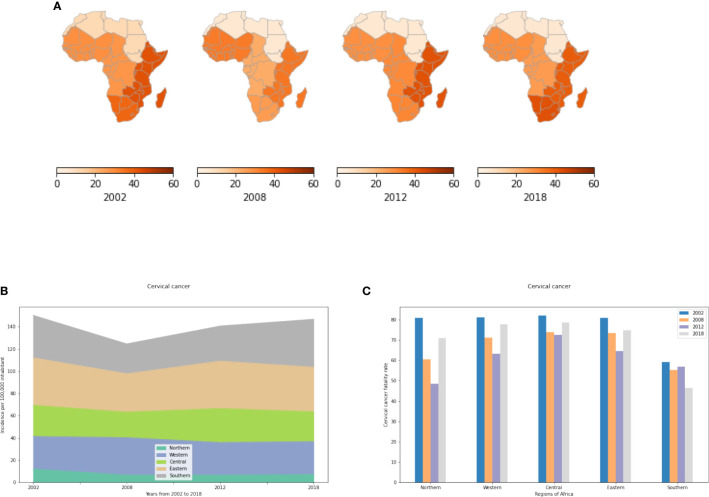
Cervical cancer incidence and fatality rates in the different African regions. **(A)** Incidence of cervical cancer per 100,000 inhabitant by African regions. **(B)** Evolution of Incidence rates from 2002 to 2018. **(C)** Fatality rates (percent mortality per year of those afflicted) of cervical cancer.

Cervical cancer is most prevalent in sub-Saharan Africa (**Figures 3A, B**). North Africa had the fewest number of cases reported in 2018 with approximately 7 cases per 100,000 women compared to 27 to 30 cases per 100,000 women in the Central and Western regions and 40 to 43 cases/100,000 women in Eastern and Southern Africa (**Table S4**). The low incidence rate in North Africa is mainly explained by advances in cervical cancer screening such as regular Papanicolaou (Pap) and human papillomavirus (HPV) DNA testing. In addition, socio-cultural and religious norms might influence sexual and reproductive health behavior in a manner reducing cervical cancer incidence rates in Northern Africa. In Eastern, Central and Western Africa, IR has decreased slightly during the last 4 years basically due to HPV vaccination programs in parts of these regions (**Supplementary Figure S1**). However, despite the HPV vaccine being used in South Africa (free HPV vaccine for schoolgirls started in March 2014) cervical cancer incidence rates in South Africa are still increasing dramatically. Therefore, other risk factors seem to contribute to cervical cancer incidence rates.

#### Cervical Cancer (FR)

Cervical Cancer, apparently the only cancer that can actually be prevented, exhibits high fatality rates in Africa. Even in 2018, more than 75% of affected women died of this cancer per year in East, Central, and West Africa. Fatality rates are decreasing only in Southern Africa. In all other African regions, fatality rates have increased during the last 4 years including Northern Africa where the incidence rate is very low but fatality rates are high (**Figure 3C**).

### Lung Cancer

Lung cancer, the most common cancer in the world for several decades, saw around 2.1 million new cases in 2018 (12). It is a highly aggressive cancer responsible for more than 1.6 million deaths per year worldwide (38). Significant decreases in lung cancer mortality rates have been observed in upper income countries due to increased awareness of the harmful effects of smoking and other risk factors (39). In contrast, lung cancer incidence and mortality rates have increased in some low and middle income countries (40). This difference is mainly due to increases in smoking (increase of tobacco, water pipes, cannabis smoking and passive smoking), as well as limited access to screening, diagnosis facilities and to appropriate targeted therapies. Several other risk factors such as asbestos exposure, dust, fumes, nickel, silica and insecticides have been reported. In Africa, there are countries that have yet to ban or restrict asbestos (39). In addition, increased life expectancies throughout Africa increase the likelihood of contracting and dying from lung cancer. Moreover, many studies have described the genetic susceptibility to develop lung cancer especially in North Africa by identifying genetic biomarkers in *EGFR*, *KRAS* and *ALK* genes (41).

**Figure 4:**
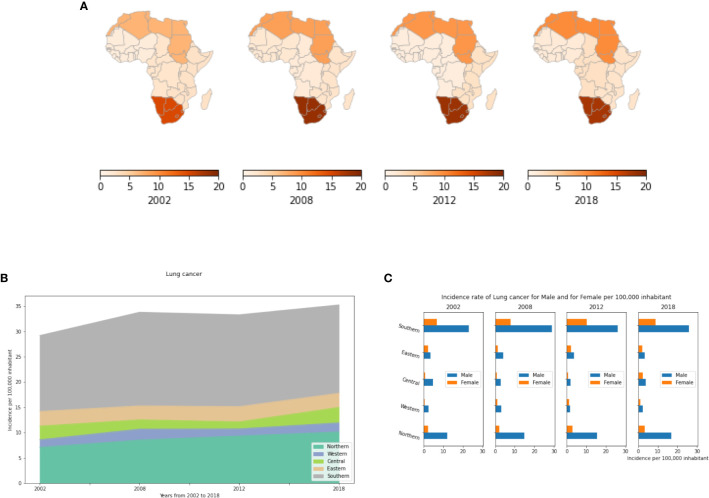
Lung cancer incidence rates by African region, per year and by gender. **(A)** Incidence rate per 100,000 inhabitant by African regions. **(B)** Dynamics of Changes of incidence rates from 2002 to 2018. **(C)** Lung cancer Incidence rates by gender in different African regions. This represents the number of active cancer cases per year per 100,000 men and per 100,000 women in each African region.

Our results show that Lung cancer is highest and on the rise in Northern and Southern Africa in both men and women (**Figures 4A, B**) mainly because of the increasing number of smokers with a prevalence of 3 to 5 fold higher among males compared to females (**Figure 4C**). The IR in Southern Africa is twice that of Northern Africa. This is likely explained by high tobacco, cannabis and alcohol use in Southern Africa. In the Eastern, Central and Western areas, the number of cases was less than 3 cases per 100,000 inhabitants in men and women combined in 2018 (**Table S5**). While currently not available in the data, for the future, a priority should be placed on distinguishing small cell lung cancer and squamous cell lung cancer (common to smokers) from non-small cell lung cancer (common to non-smokers). Such information would aid health officials with cancer sources, prevention, early detection, and public health mitigation programs.

### Stomach Cancer

Stomach cancer is the sixth most common cancer worldwide with 1,033,701 new cases reported in 2018. About half of these cases occurred in Eastern Asia. It also remains the third leading cause of cancer related deaths worldwide with a median overall survival of 9-16 months once metastatic (42). Several risk factors are involved in the development of stomach cancer including a diet high in salty and smoked foods, a diet low in fruits and vegetables, family history of stomach cancer and stomach polyps, long-term stomach inflammation, pernicious anemia, smoking, and infection with *Helicobacter pylori (H. pylori)*. *H. pylori* is a gastric pathogen that infects approximately 50% of the world’s population. Infection with *H. pylori* causes chronic inflammation and significantly increases the risk of developing duodenal and gastric ulcer disease, and gastric cancer. Africa had the highest rate of *H. pylori* infection with a prevalence of 70.1%, followed by South America and Western Asia with prevalences of 69.4% and 66.6%, respectively (43). Moreover, a family history of gastric cancer, of Lynch syndrome and of familial adenomatous polyposis, and genetic mutations mainly on the *CDH1* gene are strong risk factors known to be associated with hereditary stomach cancer.

**Figure 5:**
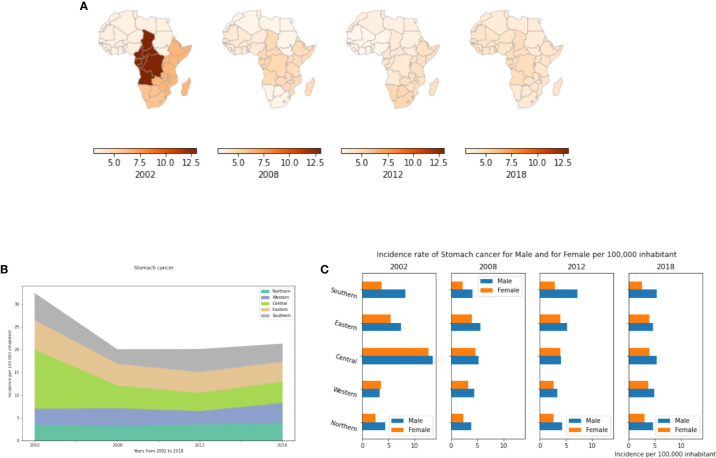
Stomach cancer incidence rates by year, by African region and by gender. **(A)** Stomach cancer incidence per 100,000 inhabitant in African regions. **(B)** Dynamics of incidence rates from 2002 to 2018. **(C)** Stomach cancer incidence by gender.

In this study, we showed that incidence rates of stomach cancer are relatively constant across African regions with a consistent gender bias as more men than women exhibit the cancer. In 2002, Central Africa far exceeded other regions with Southern and Eastern Africa showing the next highest incidence. Central Africa in 2002 had 13 cases per 100,000 population (**Figure 5A**, **Table S6**). This is mainly explained by high *H. pylori* infection rates in this region at that time. The majority of literature on *H. pylori* in Central Africa was reported from Cameroon, and similarly to other African studies, a strong association between gastritis and *H. pylori* was found (44). Ankouane and colleagues observed that 71.2% of patients with atrophic gastritis were *H. pylori* positive. The authors also found a statistically significant association between the severity of atrophic gastritis and *H. pylori* infection. Since 2008, stomach cancer incidence rates have decreased dramatically in most African regions (**Figure 5B**) with slight increases in Northern Africa. By 2018, all African regions were seeing just 3 to 5 cases per 100,000 inhabitants (**Table S6**). Our estimates still show that more cases are reported in Sub-Saharan Africa compared to Northern Africa (**Supplementary Figure S2**). **Figure 5C** shows that stomach cancer is more prevalent in males compared to females in all African regions notably in Northern and Southern Africa where its prevalence is 2 fold higher in males compared to females.

### Colorectal Cancer

Colorectal cancer is the sixth most common cancer in Africa (3, 45). At diagnosis, most cases are metastatic and in an advanced state. Consequently, fatality rates are high (46). Potential risk factors such as diet, lifestyle, socio-economic status, urbanization, Crohn’s disease, and diabetes mellitus predispose one to colorectal cancer. While arguable, prior *Schistosomiasis* infection may also be a risk factor (47). In addition, 5% of colorectal cancer cases may include underlying genetic predispositions from germline disorders such as Lynch syndrome, familial adenomatous polyposis, and mutations on genes involved in the mismatch repair pathway (48). Hereditary factors may be pronounced in Africa, since 25% of affected individuals are under the age of 40 years (45, 49).

**Figure 6:**
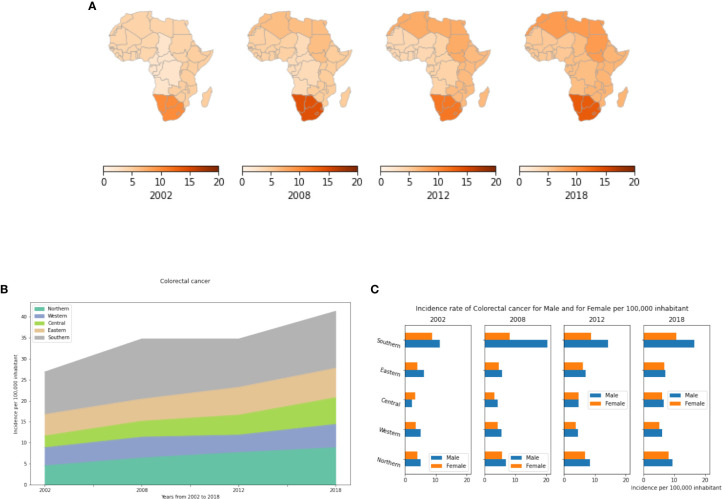
Colorectal cancer incidence rates per year, by African region and by gender. **(A)** Colorectal cancer incidence rates per 100,000 inhabitant in different African regions. **(B)** Dynamics of incidence rates from 2002 to 2018. **(C)** Colorectal cancer Incidence rate by gender in Africa.

Results presented in **Figure 6** show that incidences of colorectal cancer have been increasing since 2002 in all African regions. Southern Africa has the highest incidence followed by Northern Africa (**Figures 6A, B**). Southern Africa began with a high incidence in 2002, and since, there has been a 1.3 fold increase up to 2018. (**Table S7**). In Northern and Central Africa the incidence rates have doubled between 2002 to 2018 with 4.55 and 2.89 cases per 100,000 population in 2002 to 8.85 and 6.35 cases per 100,000 population in 2018, respectively. Except for Southern Africa where colorectal cancer is 1.2 to 2 fold more prevalent in males than in females, no significant gender differences were observed in other African regions (**Figure 6C**).

### Esophageal Cancer

Esophageal cancer (EC) is the tenth most common and the sixth most common cause of mortality among cancers worldwide. There were records of 572,034 new cases worldwide in 2018 representing 3.2% of all cancers, among them 28,494 (5.0%) were recorded from African (12). Risk factors for developing EC include smoking and chewing tobacco (50, 51), heavy consumption of alcohol (52), drinking hot beverages (53), exposure to polycyclic aromatic hydrocarbons (PAH) (54), consuming red meat (55), poor oral health (54), low intake of fresh fruits and vegetables (56), and acid reflux. Moreover, certain viruses, e.g., human papillomavirus, herpes simplex virus, cytomegalovirus, and Epstein-Barr virus, have been implicated in EC development by infecting the esophageal epithelium. Often EC manifests first as Barrett’s esophagus, which then may or may not progress to cancer. In Europe and North America, Barrett’s Esophagus is diagnosed early, monitored, and sometimes treated. Such early detection is unavailable to most Africans.

**Figure 7:**
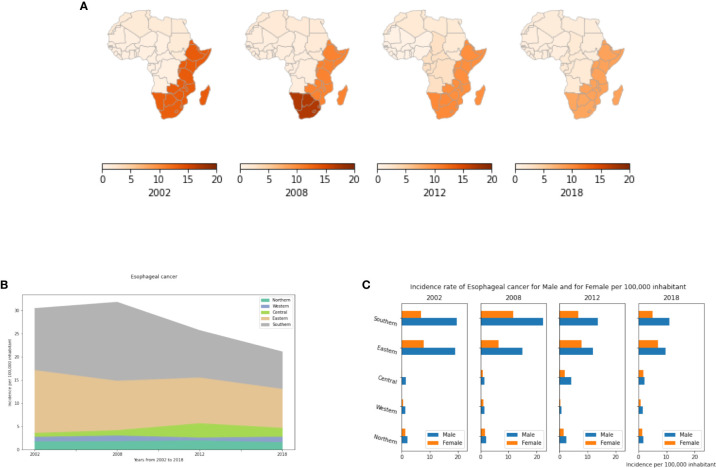
Esophageal cancer incidence rates per year, by African region and by gender. **(A)** Esophageal cancer incidence per 100,000 inhabitant in African regions. **(B)** Dynamics of incidence rates from 2002 to 2018. **(C)** Esophageal cancer incidence by gender.

Our results showed an exceptionally high prevalence of the disease in both Eastern and Southern Africa compared to other African regions (**Figure 7A**), though both of these regions have seen declines over the period from 2002 to 2018 (**Figure 7B**). In 2018, there were less than 2 cases per 100,000 inhabitants in the Northern, Central and Western regions, compared to over 8 cases per 100,000 people in Eastern and Southern Africa (**Table S8**). Like previous cancer types, analysis of all subgroups suggests that Age-standardized incidence rates of EC in Africa are generally higher in men than in women, and almost double in males compared to females in Southern and Eastern Africa (**Figure 7C**). This is mainly explained by the prevalence of tobacco and alcohol consumption that are much higher in males than females in Africa (57). However, disparities in smoking prevalence estimates have been observed between different countries and/or regions in Africa. Indeed, a recent study provided estimates of smoking prevalence and smokeless tobacco (SLT) use at the country-level and assessed their social determinants in 30 African countries. The authors showed that smoking prevalence differs significantly across African regions, which may explain the disparities in incidence rates of smoking related diseases such as cancer (58).

### Liver Cancer

Liver cancer is the seventh most common cancer worldwide, fifth in males, and ninth in females. In Africa, it is the fourth most common cancer, where its prevalence and etiology show some differences between North and sub-Saharan Africa. Despite its well-known and preventable risk factors, mortality due to this cancer remains very high. In addition, its IR are known to be significantly associated with high levels of viral infection and synergistic environmental risk factors. Viral hepatitis and the human immunodeficiency virus (HIV) are known to increase a person’s lifetime risk of liver cancer. Moreover, the rapid increase of urbanization has promoted a sharp increase in additional risk factors like coinfection, aflatoxin exposure, iron overload, type 2 diabetes mellitus and obesity.

**Figure 8:**
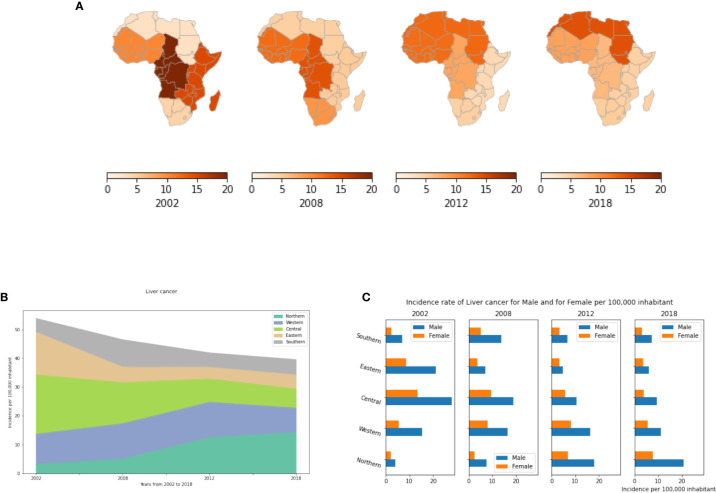
Liver cancer incidence rates by year, by African region and by gender. **(A)** Liver cancer incidence rate per 100,000 inhabitant in different African regions. **(B)** Dynamics of the incidence from 2002 to 2018. **(C)** Liver cancer incidence by gender.

Our analysis showed that while incidence of liver cancer across all of Africa has been declining since 2002, regional trends differ in striking ways (**Figure 8A**). In 2002, Central and Eastern Africa had the highest and second highest incidences, respectively. Since then, both regions have seen substantial declines. While among the lowest of regions in 2002, Northern Africa has seen an alarming increase to now being the highest among all regions. The number of diagnosed cases has risen from 3.2 in 2002 to 14.3 cases per 100,000 in 2018 (**Table S9**). The high incidence of liver cancer in North Africa is mainly due to the unusually high prevalence of hepatitis C virus (HCV) infection in Egypt. In the other African regions, liver cancer incidence rates have decreased to less than 8 cases per 100,000 population in 2018 (**Figure 8B**, **Table S9**). Males have higher incidences than females, with male-to-female ratios as high as 3:1 in some African regions such as Central and Southern Africa (**Figure 8C**). This gender difference may be linked to higher exposure to carcinogens such as tobacco and alcohol, as well as the natural protective influences of estrogen against liver inflammation (59).

### Bladder Cancer

Bladder cancer is a significant health problem. Evidence is emerging regarding gene-environment interactions associated with acquiring bladder cancer. Tobacco and occupational exposures remain the highest risk factors (60). Cigarette smokers compared to non-smokers are more likely to be diagnosed with invasive bladder cancer (61). In addition, cancer rates may be elevated in workers exposed to chemical products such as printing companies, hairdressers and truck drivers (62). Other risk factors include bladder birth defects, not drinking enough fluids, consumption of certain medicines or herbal supplements, and chronic bladder irritation and infections. Genetic risk factors associated with bladder cancer include mutations of the retinoblastoma, *RB1*, gene as well as mutations in *PTEN* that are also associated with breast and thyroid cancers and Cowden disease. People with Lynch syndrome might also have an increased risk of bladder cancer as well as other cancers of the urinary tract.

**Figure 9:**
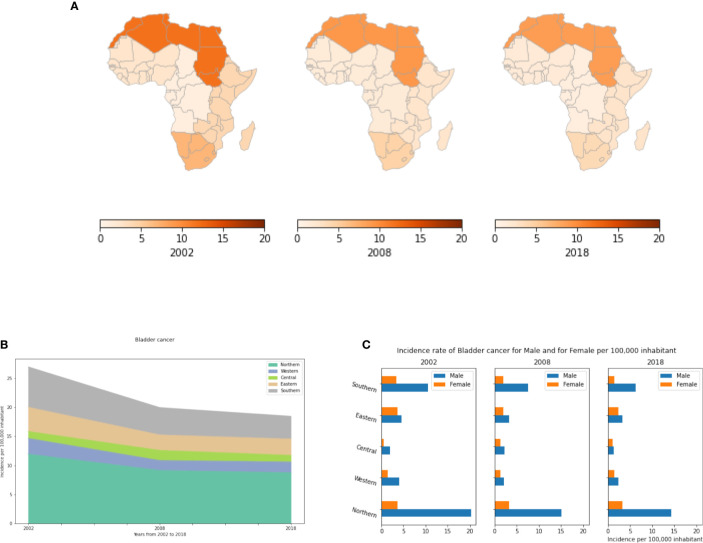
Bladder cancer incidence rates per year, by African region and by gender. **(A)** Bladder cancer incidence per 100,000 inhabitant in all Africa regions. **(B)** Dynamics of incidence rates from 2002 to 2018. **(C)** Bladder cancer incidence by gender.

In the present study, we demonstrate that in Africa, bladder cancer represents a comparatively uncommon cancer that has been declining since 2002 in all regions. Incidence rates vary significantly between regions. Northern Africa has the highest incidence (**Figures 9A, B**). This might be explained by some genetic predispositions between the different African regions, and it may be related to the very high consumption of tobacco in Northern Africa. In 2018, 8.75 cases were recorded in Northern Africa compared to less than 3.9 cases per 100,000 inhabitants in the other regions (**Table S10**). Moreover, this cancer is much more common in men than in women. In North Africa, its incidence in men is 5 fold higher than in women (**Figure 9C**). In Tunisia, bladder cancer represents the second most common cancer in males after lung cancer.

### Thyroid Cancer

Thyroid cancer develops from the tissues of the thyroid gland (63). In 2012, 298,000 new cases occurred globally. Incidence rates have increased in the last few decades, which is believed to be due to improvements in diagnostics. Globally, there were 567,233 recorded new cases and 41,071 reported deaths in 2018. Thyroid cancer most commonly manifests between the ages of 35 and 65 (64).

Several risk factors have been proven to be associated with thyroid cancer. The most studied and proven risk factors being radiation exposure. Sources of such radiation include certain medical treatments as well as radiation fallout from power plant accidents or nuclear weapons. Other risk factors include being overweight and having a diet low in iodine. Although the genetic component of thyroid cancer is still not well defined, several hereditary forms have been identified including:

- Familial medullary thyroid carcinoma (FMTC). FMTC can occur alone, or it can be seen along with other tumors caused by mutations in the *RET* gene.
- People with Familial adenomatous polyposis (FAP) known to develop many colon polyps and or colon cancer also have a very high risk of developing papillary thyroid cancer.
- People with Cowden disease have an increased risk of thyroid problems and certain benign growths (including some called hamartomas). The thyroid cancers tend to be either the papillary or follicular type. This syndrome is most often caused by mutations in the *PTEN* gene.
- People with Carney complex, type I may develop a number of benign tumors and hormone problems. They also have an increased risk of papillary and follicular thyroid cancers. This syndrome is caused by mutations in the *PRKAR1A* gene.
- Familial non medullary thyroid carcinoma: genes on chromosome 19 and chromosome 1 are suspected of causing these familial cancers.

Moreover, like other cancer types, the number of cancer cases and mortality rates differ between populations. Those of Asian ancestry exhibit higher incidences (65, 66).

**Figure 10:**
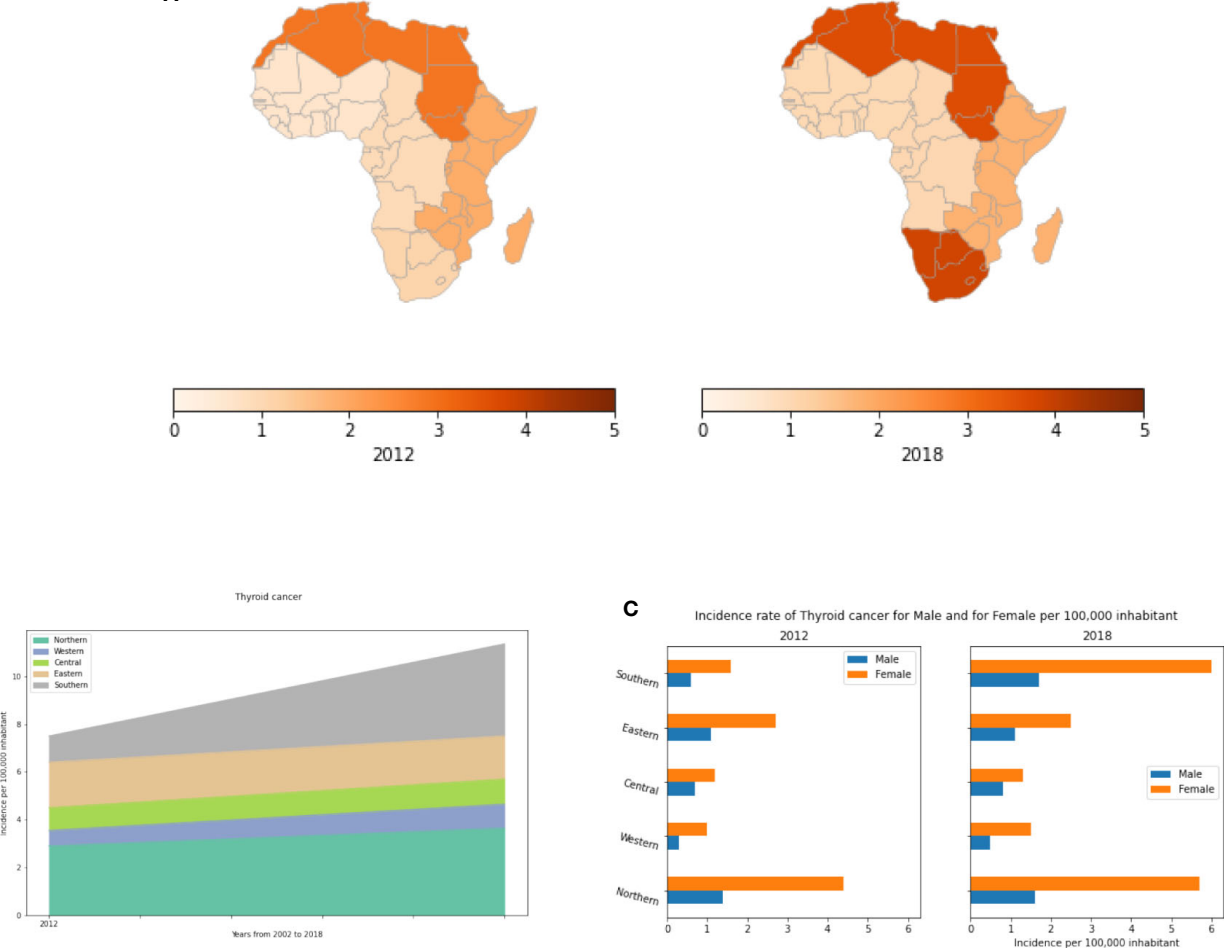
Thyroid cancer incidence rates per year, by African region and by gender. **(A)** Incidence rates per 100,000 inhabitant in different African regions. **(B)** Dynamics of the incidence from 2012 to 2018. **(C)** Thyroid cancer incidence rates by gender in Africa.

In Africa, thyroid cancer is a rare. In 2002, Northern and Eastern Africa had the highest incidences (**Figures 10A, B**). While most regions remained relatively stable in their incidence rates, from 2002 to 2018, Northern and Southern Africa had increases in the number of thyroid cancer cases. The number of cases has increased 3.5 fold between 2012 and 2018 in Southern Africa, and 1.25 fold in Northern Africa (**Table S11**). Unlike other cancer types, thyroid cancer is much more common in women than in men (**Figure 10C**). In 2018, the number of affected women was 2-3 fold higher than men in almost all African regions.

### Available Medical Devices

**Figure 11:**
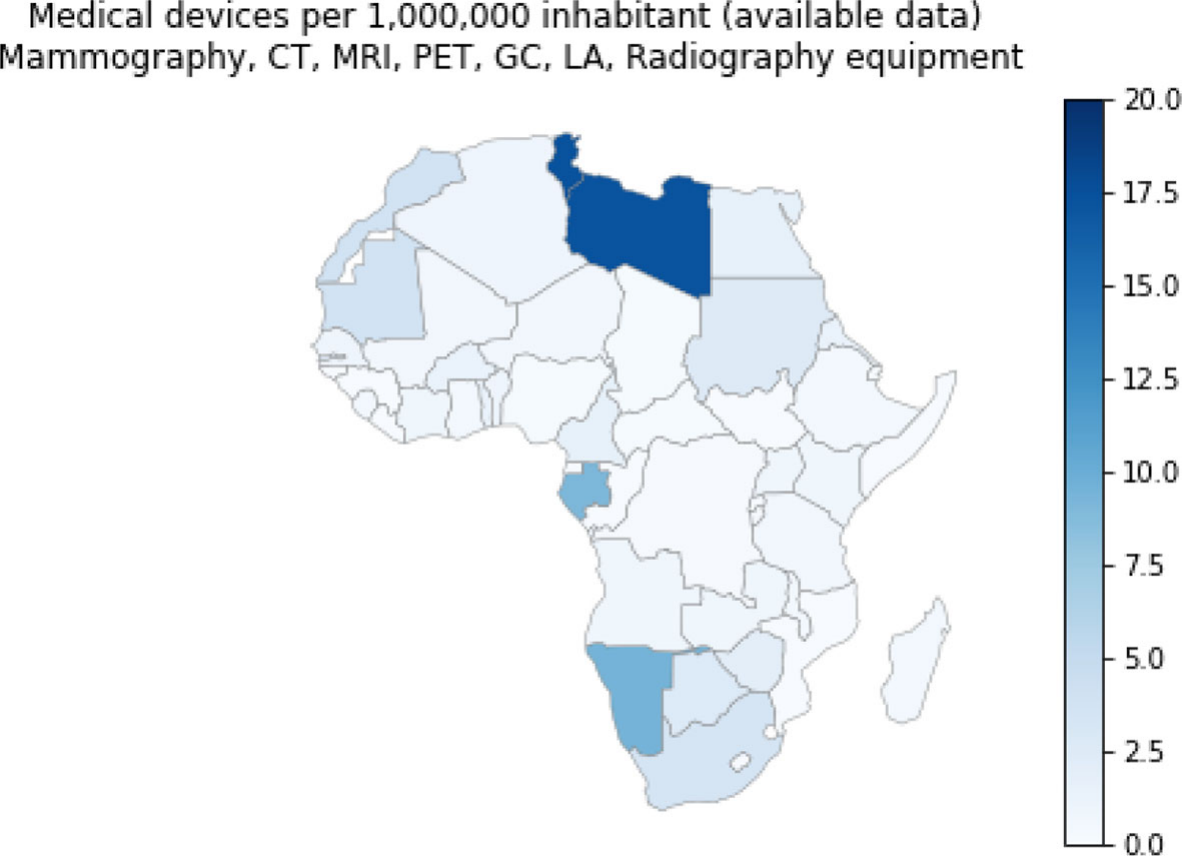
Cancer Medical devices per 1,000,000 inhabitants. These include the following medical devices: Mammographs, Computed Tomography, Magnetic Resonance Imaging, Positron Emission Tomography, Gamma Camera or Nuclear Medicine, Linear accelerator, Telecobalt unit, Radiotherapy. Source Global atlas of medical devices, World Health Organization, 2017.

We extracted data on the list and availability of medical devices in each African country from the Global atlas of medical devices provided by the World Health Organization (2017). This data includes statistics on national policy on health technology, medical device incorporation, inventory and maintenance, lists of medical devices, healthcare facilities per 100,000 population, and medical equipment per 1,000,000 population. **Figure 11** shows the distribution of the following cancer medical devices in Africa: mammographs, computed Tomography (CT, a three-dimensional imaging method using x-rays to scan body areas slice-by-slice), gamma camera (also called Anger camera or scintillation camera used in nuclear medicine for the visualization of physiological or biochemical functions in the body), Magnetic Resonance Imaging (MRI, a 3-D imaging method well suited for soft tissue diagnostics), Positron Emission Tomography (PET, an imaging method for diagnostics in nuclear medicine using positron-emitting radionuclides), and radiotherapy equipment (using ionizing radiation to control or destroy malignant cells). Data on these medical devices are not available for all African countries. The best equipped countries, according to the available data per 1,000,000 inhabitant, are in descending order: Seychelles (33.3), Mauritius (24.5), Tunisia (17.4), Libya (17.3), Cape Verde (14.9), Namibia (9.5) and Gabon (9.1). Countries with either little equipment or missing data include: Liberia, Mozambique, Lesotho, Guinea-Bissau, Guinea, Rwanda, Equatorial Guinea, Djibouti, Democratic Republic of the Congo, Sao Tome and Principe, South Sudan and Somalia (**Figure 11**). If we assume that the lack of data from a country correlates with a lack of equipment, then we believe that the country by country map in **Figure 11** provides an ordinal but not absolute scale of availability. And, if so, we see regional trends where northern and southern Africa have the highest concentration of equipment while central Africa has the lowest where Gabon provides a notable exception.

### Human Development Index (HDI) and Cancer (Breast, Prostate, and Cervical)

**Figure 12:**
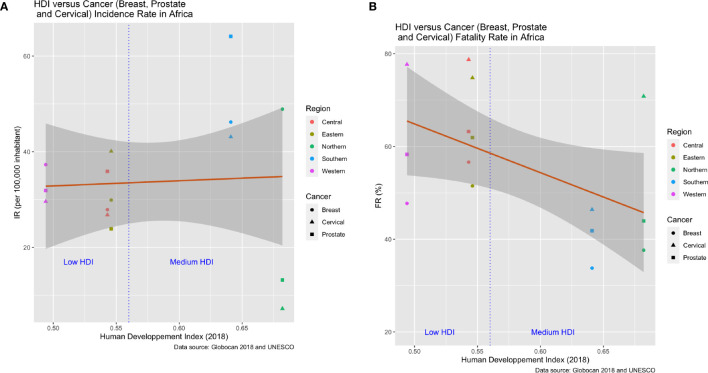
Association between human development index (HDI) and cancer incidence and fatality rates for the five African regions in 2018. The data include fifteen points per graph resulting from three cancer types (breast, prostate and cervical) per region. **(A)** The relationship between HDI and cancer incidence rates is not significant and shows no trends other than higher variance among the cancer types for the two regions (Northern and Southern Africa) with the highest HDI. **(B)** Cancer fatality rates decline significantly with HDI for the three most frequent cancer types.

We used least-squares linear regression analyses to test for correlation between the HDI and the incidence (IR) and fatality rates (FR) of the three most common cancer types (breast, prostate and cervical) in the five African regions (**Figure 12**). This generated 15 data points for each analysis: 3 cancers by five regions. There was no detectable relationship between HDI and incidence, though the scatter among cancer types is much higher for the two regions (Northern and Southern Africa) with the highest HDIs. Fatality rates decline significantly with HDI. If we classify Northern and Southern Africa as medium HDI, and the remaining three regions as low HDI, then the striking difference concerns the significantly higher fatality rates for the low HDI regions compared to the medium HDI ones. HDI likely correlates with early detection and a broader range of therapy options for those burdened with cancer. We add two caveats to these results. First, countries can vary strikingly in HDI within regions, though overall, high HDI countries are clustered in northern and southern Africa. Second, country by country reporting of variables comprising the HDI may have discrepancies. Placed in these contexts the results are intriguing, tentative and deserving of follow-up.

## Discussion

Population origin and diversity are known to influence cancer incidence, survival, drug response, molecular pathways, and ultimately the treatment outcome (67). Although these factors differ widely among human populations, most genetic and epidemiological cancer studies and discoveries have been reported on non-African populations, particularly those of European descent (68). Appropriately, much effort in Africa has been invested towards managing and curing communicable diseases such as Malaria, Tuberculosis and HIV. However, cancer has received much less attention even as incidences and mortality from the various cancer types are generally increasing continent-wide. For cancer in Africa, little is known regarding its epidemiology, specific risk factors, and genetic components, particularly in terms of how Africa may differ from Western countries. Africa seems noteworthy for the large proportion of young patients and aggressive forms of the disease (69). Additionally, Africans continue to face a burden of biotic (e.g., infectious) and abiotic factors as well as lifestyle changes that impact cancer susceptibility and outcome. In terms of pan-African trends in prevalence, thyroid, colorectal, lung, prostate and breast cancer rates have been trending upwards from 2002 to 2018. Cervical and Stomach cancers have remained relatively stable. Incidences of bladder, liver and esophageal cancers have declined. Pan-African fatality rates for cervical, breast and prostate cancer have mostly been trending downwards from 2002 to 2018. In 2018, fatality rates from cervical, breast and prostate cancer hovered around or above 50%, 40% and 30%, respectively, across the five regions. As expected, there is considerable region to region variability in incidences and fatality rates.

**Figure 13:**
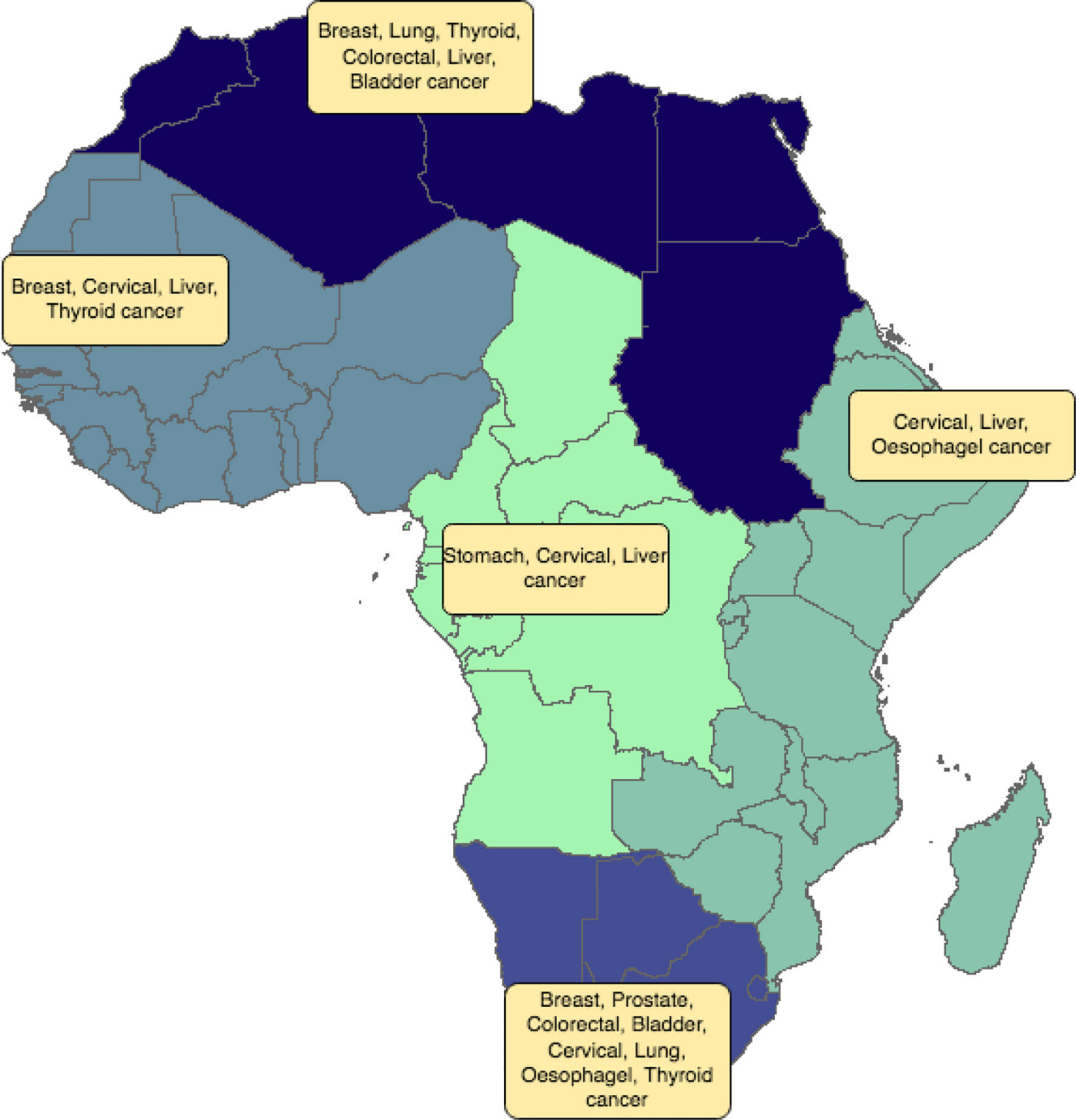
Distribution of the most frequent cancer types by African region. Different colors refer to the different African regions (North, East, West, Central and Southern Africa).

In this study we show some of the disparities in cancer incidence and fatality rates that exist between the different African regions. In general, Southern Africa is a high-incidence area with a pattern of risk factors similar to those identified in Northern Africa. Recent decades have seen many lifestyle changes for Southern and Northern Africans, including urbanization, adopting of Western lifestyle habits, and increasing tobacco and alcohol consumption. Availability of diagnostic equipment and screening methods in Southern and Northern Africa can also identify cases and raise incidence rates. Hence, lower incidences in Central, Eastern and Western Africa may be more apparent than real as a result of failures to diagnose. Not all African countries have the same facilities in terms of screening and disease detection. This will influence the degree to which a country unintentionally under-reports actual incidence rates. In this context, our results also showed a significant association between Human Development Index and cancer fatality rates. For most cancer types, fatality rates were lower in Northern and Southern Africa compared to other African regions. These differences can stem from under-diagnosis of actual incidence rates, the severity of the disease at diagnosis, and access to therapy. Three major risk factors seem to influence cancer incidence rates in Africa: environmental factors, genetics and infectious agents. Consequently, changing lifestyle habits, having access to genetic testing, and vaccination would help decrease incidence rates. In Northern and Southern Africa, the most frequent cancers are those observed in Western countries (breast, colon, and prostate). This pattern differs from that of other African regions and countries, where infection-related cancers predominate (**Figure 13**). Early detection and better access to more diverse treatment options would likely bring down fatality rates.

In Southern and Northern Africa with very high incidences of prostate, colon and breast cancer, early detection and some lifestyle shifts could allow for early cure and lower incidences, respectively. To reduce IR and FR of these three cancer types, health systems could apply accurate genetic testing, and use newer targeted and immunotherapies, as these two regions have some access to the most up-to-date technologies and therapies. In Western, Central and Eastern regions, cervical cancer, a highly preventable cancer, creates a large public health burden. Therefore, we support widely available vaccinations and campaigns to increase awareness of the links between sexual behavior and cervical cancer. The high incidence of some specific cancers in certain regions remains largely unexplained. For instance, esophageal cancer is much more prevalent in Eastern Africa than in any other region. The prevalence of bladder and thyroid cancer in Northern Africa seems anomalous. We advocate epidemiological studies on cancer risk factors tailored specifically for each African region (or country), and relevant cancer types. Such studies are necessary to understand the conditions that have created the perfect storm that drives disparities in cancer outcomes inside Africa. The goal should be country- and region-specific plans to reduce cancer incidences and mortality among Africans.

Except for thyroid cancer, our results reveal higher incidences of the remaining cancer types in males compared to females (not withstanding breast and cervical cancer). Sometimes the difference is 2-5 fold! Some of this difference may be attributable to smoking, alcohol consumption and exposure to environmental carcinogens at work or outside of the home. Higher male incidence than females has also been observed in non-African populations. A portion of the difference may be the absence of a second X chromosome in males (70). showed that a subset of X-chromosome tumor suppressor genes can escape from X-inactivation that might occur from a gene mutation on one of the X-chromosomes. The authors conclude that biallelic expression of these genes in females explains a portion of the reduced cancer incidence compared to males across a variety of cancer types. How large this effect is should be studies and remains unknown.

Our work reveals unanswered questions regarding cancer epidemiology and genetics in Africa. Therefore, we highly recommend African governments, policy makers, and international organizations such as the World Health Organization direct efforts towards cancer research that will improve decision making, and improve the health of African populations. For Africa, cancer research is a necessity, not a luxury. Indeed, very few or no resources are allocated for cancer research in Africa, and very few data are available on medical devices used in cancer care. Much can be gained by better and more comprehensive record keeping. When collated, curated, and stored electronically such data can identify patterns, and opportunities for interventions. Indeed, a limitation of our analyses rests on making estimates of incidence and fatality rates, HDI, and medical devices from the Global Cancer Observatory database, UNESCO, and WHO. While the best sources currently available, their representation of the data relies on the representativeness and quality of the source information as gleaned from or provided by individual countries and their Health Ministries. Therefore, country or continent-wide data repositories can direct research towards cancers and regions where most needed. Healthcare, clinical and epidemiological research allow for evidence based formulations of health policies and allocations of scarce resources towards facilities, diagnostics and therapeutics. The lack of evidence based decision making in Africa squanders opportunities related to cancer research and cancer care (71).

Untapped opportunities exist to reduce the burden and disparities due to cancer by enhancing cancer communication and public health messages about affordable care and prevention strategies and by expanding the targets of engagement to include private sector stakeholders, researchers, epidemiologists, learned societies and advocacy groups.

As in the case of cervical cancer, the fatality rates of all cancers in Africa will be influenced by sociocultural, religious and gender norms. Such norms will vary across regions and between countries. Particular norms will influence cancer screening, a person’s ability or willingness to seek treatment, and health disparities, This points to the need to include social scientists, social workers and diverse public health officials in taking broad-based approaches to improving cancer prevention and outcomes. Future work could include evaluating the role of norms in facilitating or hindering cancer care in Africa.

## Conclusion

Cancer has received low priority for health care services in Sub-Saharan Africa. This study shows that there are several disparities in cancer diagnosis and screening between the different African regions that can be one of the reasons for differences in cancer incidence and mortality rates across regions. There are pending concerns regarding cancer care in the continent. Therefore, Africa has to invest in cancer prevention, management and in evidence-based care. Investing in cancer research will help to understand risk factors specific to Africa or to specific regions of Africa. The improvement of cancer clinical care in Africa can be achieved by making evidence based decisions using key indicators including metrics on urbanization, HDI, co-morbidity, availability of medical devices, vaccination and life expectancy. Cancer incidence data need to be evaluated at the national and regional level by implementing accurate cancer control programs. The relative advances in cancer screening and diagnosis in Southern and Northern Africa can be taken as a model for other African countries and regions.

## Data Availability Statement

The original contributions presented in the study are included in the article/**Supplementary Material**, further inquiries can be directed to the corresponding author.

## Author Contributions

YH, IA-T, and AB designed the workflow and wrote this paper. AZ and IA-T collected the data and analyzed it. SA, SB, and JB revised the manuscript. All authors contributed to the article and approved the submitted version.

## Conflict of Interest

The authors declare that the research was conducted in the absence of any commercial or financial relationships that could be construed as a potential conflict of interest.

## Footnotes

1 Density per 1,000,000 females aged from 50-69 old. Several countries have adopted breast cancer screening programs as an effective way for early detection of the disease, using tools such as mammography machines (17). This indicator shows the number of dedicated mammography machines (those designed exclusively for taking mammograms) available in Africa. From the age pyramids of the various African countries (https://www.populationpyramid.net/), the data of Mammographs per 1,000,000 inhabitants was adjusted. This adjustment involved multiplying the number of mammographs (data available are the density per 1,000,000 females aged from 50-69 old) by the population percentage of women between 50 and 69 years old, see **Table S13** and **Figure S3**.
