# Supplementary material for: Cancer in Africa: The Untold Story
Source: Front Oncol. 2021 Apr 15;11:650117. doi: 10.3389/fonc.2021.650117 (PMC8082106; doi:10.3389/fonc.2021.650117)
Supplement: Supplementary file 1 [file DataSheet_1.pdf]

*Supplementary Material*

## SUPPLEMENTARY TABLES

| **Northern Africa (210,002,000)** | **Western Africa (331,255,000)** | **Central Africa (135,750,000)** | **Eastern Africa (373,202,000)** | **Southern Africa (60,425,000)** |
|---|---|---|---|---|
| Morocco (35,280,451) | Mauritania (4,166,000) | Chad (14,485,740) | Eritrea (5,351,000) | Namibia (2,513,000) |
| Algeria (40,610,154) | Senegal (15,580,485) | Central African Rep. (4,998,000) | Ethiopia (101,850,000) | Botswana (23,003,000) |
| Tunisia (11,411,212) | Gambia (2,054,000) | Cameron (23,920,400) | Uganda (41,175,440) | South Africa (54,975,900) |
| Libya (6,330,000) | Guinea-Bissau (1,888,000) | Eq. Guinea (869,000) | Kenya (47,450,250) | eSwatini (1,220,000) |
| Egypt (93,380,570) | Guinea (12,940,000) | Gabon (1,763,000) | Djibouti (899,000) | Lesotho (2,160,000) |
| Sudan (41,175,440) | Sierra Leone (6,126,000) | Congo (4,740,000) | Somalia (11,079,000) | |
| South Sudan (12,700,000) | Liberia (4,615,000) | Dem. Rep. Congo (81,331,050) | Rwanda (11,880,000) | |
| | Côte d'Ivoire (23,250,180) | Angola (25,830,975) | Burundi (11,552,000) | |
| | Mali (18,130,825) | Sao Tomé-et-Principe (194,000) | Tanzania (55,155,470) | |
| | Burkina Faso (18,630,825) | | Malawi (17,730,820) | |
| | Ghana (28,033,375) | | Zimbabwe (15,960,810) | |
| | Togo (7,496,000) | | Zambia (16,770,330) | |
| | Benin (11,166,000) | | Madagascar (24,815,920) | |
| | Niger (20,715,820) | | Mozambique (23,700,715) | |
| | Nigeria (186,987,000) | | Seychelles (97,000) | |
| | Sahara Occidental | | Comoros (807,000) | |
| | Cap-Vert (526,000) | | | |

**Table S1.** List of the African regions, countries and population (Source: World Health Organization https://www.who.int/choice/demography/african_region/en/).

| | 2002 | 2008 | 2012 | 2018 |
|---|---|---|---|---|
| Northern | 23.2 | 32.7 | 43.2 | 48.9 |
| Western | 27.8 | 31.8 | 38.6 | 37.3 |
| Central | 16.5 | 21.3 | 26.8 | 27.9 |
| Eastern | 19.5 | 19.3 | 30.4 | 29.9 |
| Southern | 33.4 | 38.1 | 38.9 | 46.2 |

**Table S2.** Breast cancer incidence rates per 100,000 inhabitant.

| | 2002 | 2008 | 2012 | 2018 |
|---|---|---|---|---|
| Northern | 5.8 | 8.1 | 10.6 | 13.2 |
| Western | 19.3 | 22.2 | 25.1 | 31.9 |
| Central | 24.5 | 16.4 | 27.0 | 35.9 |
| Eastern | 13.8 | 14.5 | 23.3 | 23.9 |
| Southern | 40.5 | 53.9 | 61.8 | 64.1 |

**Table S3.** Prostate cancer incidence rates per 100,000 inhabitant.

| | 2002 | 2008 | 2012 | 2018 |
|---|---|---|---|---|
| Northern | 12.1 | 6.6 | 6.6 | 7.2 |
| Western | 29.3 | 33.7 | 29.3 | 29.6 |
| Central | 28.0 | 23.0 | 30.6 | 26.8 |
| Eastern | 42.7 | 34.5 | 42.7 | 40.1 |
| Southern | 38.2 | 26.8 | 31.5 | 43.1 |

**Table S4.** Cervical cancer incidence rates per 100,000 inhabitant.

| | 2002 | 2008 | 2012 | 2018 |
|---|---|---|---|---|
| Northern | 12.0 | 14.9 | 15.6 | 16.9 |
| Western | 2.4 | 3.1 | 1.7 | 2.4 |
| Central | 4.7 | 2.8 | 2.0 | 3.8 |
| Eastern | 3.6 | 4.1 | 3.8 | 3.4 |
| Southern | 23.1 | 29.0 | 26.1 | 26.0 |

**Table S5.** Lung cancer incidence rates per 100,000 inhabitant.

| | 2002 | 2008 | 2012 | 2018 |
|---|---|---|---|---|
| Northern | 3.45 | 3.15 | 3.50 | 3.85 |
| Western | 3.50 | 3.90 | 2.95 | 4.35 |
| Central | 13.00 | 5.00 | 4.00 | 4.70 |
| Eastern | 6.45 | 4.80 | 4.55 | 4.35 |
| Southern | 5.95 | 3.15 | 5.05 | 4.00 |

**Table S6.** Stomach cancer incidence rates per 100,000 inhabitant.

| | 2002 | 2008 | 2012 | 2018 |
|---|---|---|---|---|
| Northern | 4.55 | 6.40 | 7.70 | 8.85 |
| Western | 4.30 | 4.95 | 4.15 | 5.55 |
| Central | 2.80 | 3.80 | 4.75 | 6.35 |
| Eastern | 5.10 | 5.25 | 6.60 | 7.00 |
| Southern | 10.10 | 14.30 | 11.50 | 13.55 |

**Table S7.** Colorectal cancer incidence rates per 100,000 inhabitant.

| | 2002 | 2008 | 2012 | 2018 |
|---|---|---|---|---|
| Northern | 1.75 | 1.80 | 1.95 | 1.55 |
| Western | 0.95 | 1.20 | 0.60 | 1.20 |
| Central | 0.85 | 1.15 | 3.10 | 1.90 |
| Eastern | 13.55 | 10.65 | 9.85 | 8.40 |
| Southern | 13.35 | 17.00 | 10.20 | 8.05 |

**Table S8.** Esophageal cancer incidence rates per 100,000 inhabitant.

| | 2002 | 2008 | 2012 | 2018 |
|---|---|---|---|---|
| Northern | 3.20 | 5.00 | 12.50 | 14.30 |
| Western | 10.45 | 12.30 | 12.25 | 8.40 |
| Central | 20.60 | 14.25 | 8.10 | 6.65 |
| Eastern | 14.85 | 5.40 | 4.05 | 4.90 |
| Southern | 4.75 | 9.50 | 5.00 | 5.30 |

**Table S9.** Liver cancer incidence rates per 100,000 inhabitant.

| | 2002 | 2008 | 2018 |
|---|---|---|---|
| Northern | 11.95 | 9.15 | 8.75 |
| Western | 2.70 | 1.70 | 1.85 |
| Central | 1.20 | 1.75 | 1.15 |
| Eastern | 4.15 | 2.65 | 2.80 |
| Southern | 6.95 | 4.70 | 3.90 |

**Table S10.** Bladder cancer incidence rates per 100,000 inhabitant.

| | 2012 | 2018 |
|---|---|---|
| Northern | 2.90 | 3.65 |
| Western | 0.65 | 1.00 |
| Central | 0.95 | 1.05 |
| Eastern | 1.90 | 1.80 |
| Southern | 1.10 | 3.85 |

**Table S11.** Thyroid cancer incidence rates per 100,000 inhabitant.

| Region | HDI | Prostate Cancer | | Breast Cancer | | Cervical Cancer | |
|---|---|---|---|---|---|---|---|
| | | FR (%) | IR | FR (%) | IR | FR (%) | IR |
| Northern | 0.682 | 43.94 | 13.2 | 37.63 | 48.9 | 70.83 | 7.2 |
| Western | 0.494 | 58.31 | 31.9 | 47.72 | 37.3 | 77.70 | 29.6 |
| Central | 0.543 | 63.23 | 35.9 | 56.63 | 27.9 | 78.73 | 26.8 |
| Eastern | 0.546 | 61.92 | 23.9 | 51.51 | 29.9 | 74.81 | 40.1 |
| Southern | 0.641 | 41.81 | 64.1 | 33.77 | 46.2 | 46.40 | 43.1 |

**Table S12.** Human Development Index (HDI), fatality rates FR(%) and incidence (IR) of the three most common cancer types (breast, prostate and cervical) in the five African regions. Country specific HDI data were downloaded from UNESCO (http://uis.unesco.org/).

| Country | Women aged 50-69 years (percentage of population) | Mammograph | Country | Women aged 50-69 years (percentage of population) | Mammograph |
|---|---|---|---|---|---|
| Algeria | 6.6 | 0.00 | Angola | 3.4 | 6.33 |
| Benin | 4.3 | 16.01 | Botswana | 5.8 | 19.10 |
| Burkina Faso | 3.9 | 13.59 | Burundi | 3.7 | 2.69 |
| Cabo Verde | 6.3 | 140.6 | Cameroon | 3.7 | 17.4 |
| Central Afr. Rep. | 4.2 | 4.68 | Chad | 3.4 | 4.67 |
| Comoros | 4.5 | 31.27 | Congo | 4.3 | 0.00 |
| Côte d'Ivoire | 3.8 | 0.00 | Dem. Rep. Congo | 5.1 | 0.74 |
| Djibouti | 5.5 | 0.00 | Egypt | 6.2 | 0.00 |
| Eq. Guinea | 3.4 | 0.00 | Eritrea | 4.7 | 16.56 |
| Ethiopia | 4.2 | 0.00 | Gabon | 4.2 | 73.12 |
| Gambia | 3.6 | 16.49 | Ghana | 4.8 | 0.00 |
| Guinea | 4.8 | 0.00 | Guinea-Bissau | 4.4 | 0.00 |
| Kenya | 4.4 | 6.83 | Lesotho | 6.1 | 0.00 |
| Liberia | 4.5 | 0.00 | Libya | 5.5 | 0.00 |
| Madagascar | 4.3 | 6.23 | Malawi | 3.7 | 0.00 |
| Mali | 3.6 | 5.42 | Mauritania | 4.5 | 22.42 |
| Mauritius | 12.1 | 49.68 | Morocco | 8.1 | 18.46 |
| Mozambique | 4.1 | 0.00 | Namibia | 5.1 | 42.3 |
| Niger | 3.5 | 10.94 | Nigeria | 4.2 | 0.00 |
| Rwanda | 4.7 | 0.00 | Sao T. and P. | 4.4 | 0.00 |
| Senegal | 4.4 | 5.19 | Seychelles | 9.2 | 127.73 |
| Sierra Leone | 4.4 | 0.00 | Somalia | 3.8 | 0.00 |
| South Africa | 7,00 | 7.78 | S. Sudan | 4.4 | 0.00 |
| Sudan | 4.6 | 12.18 | Swaziland | 3.8 | 33.62 |
| Tanzania | 3.9 | 6.15 | Togo | 4.4 | 10.39 |
| Tunisia | 9.2 | 22.58 | Uganda | 3.2 | 4.41 |
| Zambia | 3.3 | 4.56 | Zimbabwe | 3.9 | 6.89 |

**Table S13.** The density of 1,000,000 women aged 50-69 years in African countries was extracted from https://www.populationpyramid.net/ (percentage of Women aged 50-69 years in the total population), and the density of Mammographs per 1,000,000 females aged from 50-69 old, per country, was extracted from the World Atlas of Medical Devices (World Health Organization, 2017). The density of Mammographs per 1,000,000 inhabitants has been adjusted according to the age pyramids of the various African countries: the number of mammographs (MAM) has been multiplied by the percentage of women aged between 50 and 69 years in the population (Women Density divided by 100), see Tables S14, S15, S16, S17 and S18.

| Country | MAM | CT | MRI | PET | GC | LA | R | TU | Total |
|---|---|---|---|---|---|---|---|---|---|
| Central African Rep. | 0.20 | 0.00 | 0.00 | 0.00 | 0.00 | 0.00 | 0.00 | 0.00 | 0.2 |
| Dem. Rep. Congo | 0.04 | 0.07 | 0.00 | 0.00 | 0.01 | 0.01 | 0.01 | 0.00 | 0.1 |
| Gabon | 3.10 | 3.59 | 1.20 | 0.00 | 0.00 | 0.00 | 0.60 | 0.60 | 9.1 |
| Congo | 0.00 | 0.00 | 0.00 | 0.00 | 0.00 | 0.00 | 0.00 | 0.00 | 0.0 |
| Sao Tome and Principe | 0.00 | 0.00 | 0.00 | 0.00 | 0.00 | 0.00 | 0.00 | 0.00 | 0.0 |
| Chad | 0.16 | 0.08 | 0.00 | 0.00 | 0.00 | 0.00 | 0.00 | 0.00 | 0.2 |
| Cameroon | 0.64 | 0.63 | 0.04 | 0.00 | 0.04 | 0.00 | 0.13 | 0.13 | 1.6 |
| Eq. Guinea | 0.00 | 0.00 | 0.00 | 0.00 | 0.00 | 0.00 | 0.00 | 0.00 | 0.0 |
| Angola | 0.22 | 0.42 | 0.05 | 0.00 | 0.00 | 0.00 | 0.05 | 0.05 | 0.8 |

**Table S14.** Cancer Medical devices per 1,000,000 inhabitants in Central African region: Mammographs (MAM), Computed Tomography (CT), Magnetic Resonance Imaging (MRI), Positron Emission Tomography (PET), Gamma Camera or Nuclear Medicine (GC), Linear accelerator (LA), Radiotherapy (R) and Telecobalt unit (TU). Source Global atlas of medical devices, World Health Organization, 2017.

| Country | MAM | CT | MRI | PET | GC | LA | R | TU | Total |
|---|---|---|---|---|---|---|---|---|---|
| Eritrea | 0.78 | 0.32 | 0.16 | 0.00 | 0.00 | 0.00 | 0.00 | 0.00 | 1.3 |
| Mozambique | 0.00 | 0.00 | 0.00 | 0.00 | 0.00 | 0.00 | 0.00 | 0.00 | 0.0 |
| Kenya | 0.30 | 0.25 | 0.16 | 0.00 | 0.05 | 0.00 | 0.02 | 0.02 | 0.8 |
| Zambia | 0.15 | 0.21 | 0.07 | 0.00 | 0.07 | 0.07 | 0.14 | 0.07 | 0.8 |
| Mauritius | 6.01 | 6.43 | 4.82 | 0.00 | 2.41 | 0.80 | 2.41 | 1.61 | 24.5 |
| Ethiopia | 0.00 | 0.36 | 0.07 | 0.00 | 0.01 | 0.00 | 0.02 | 0.02 | 0.5 |
| Rwanda | 0.00 | 0.00 | 0.00 | 0.00 | 0.00 | 0.00 | 0.00 | 0.00 | 0.0 |
| Zimbabwe | 0.27 | 0.42 | 0.28 | 0.00 | 0.28 | 0.21 | 0.42 | 0.21 | 2.1 |
| Djibouti | 0.00 | 0.00 | 0.00 | 0.00 | 0.00 | 0.00 | 0.00 | 0.00 | 0.0 |
| Madagascar | 0.27 | 0.13 | 0.00 | 0.00 | 0.00 | 0.00 | 0.04 | 0.04 | 0.5 |
| Somalia | 0.00 | 0.00 | 0.00 | 0.00 | 0.00 | 0.00 | 0.00 | 0.00 | 0.0 |
| Comoros | 1.41 | 1.36 | 0.00 | 0.00 | 0.00 | 0.00 | 0.00 | 0.00 | 2.8 |
| Burundi | 0.10 | 0.20 | 0.00 | 0.00 | 0.00 | 0.00 | 0.00 | 0.00 | 0.3 |
| Tanzania | 0.24 | 0.12 | 0.04 | 0.02 | 0.08 | 0.00 | 0.06 | 0.06 | 0.6 |
| Uganda | 0.14 | 0.45 | 0.08 | 0.00 | 0.05 | 0.00 | 0.05 | 0.05 | 0.8 |
| Seychelles | 11.75 | 10.77 | 10.77 | 0.00 | 0.00 | 0.00 | 0.00 | 0.00 | 33.3 |
| Malawi | 0.00 | 0.31 | 0.06 | 0.00 | 0.00 | 0.00 | 0.00 | 0.00 | 0.4 |

**Table S15.** Cancer Medical devices per 1,000,000 inhabitants in Eastern African region.

| Country | MAM | CT | MRI | PET | GC | LA | R | TU | Total |
|---|---|---|---|---|---|---|---|---|---|
| S. Sudan | 0.00 | 0.00 | 0.00 | 0.00 | 0.00 | 0.00 | 0.00 | 0.00 | 0.0 |
| Sudan | 0.56 | 1.13 | 0.32 | 0.00 | 0.13 | 0.08 | 0.18 | 0.11 | 2.5 |
| Morocco | 1.50 | 1.21 | 0.36 | 0.00 | 0.00 | 0.33 | 0.39 | 0.06 | 3.8 |
| Tunisia | 2.08 | 8.91 | 2.00 | 0.00 | 1.18 | 0.64 | 1.64 | 1.00 | 17.4 |
| Algeria | 0.00 | 0.00 | 0.00 | 0.00 | 0.00 | 0.18 | 0.43 | 0.26 | 0.9 |
| Egypt | 0.00 | 0.00 | 0.00 | 0.00 | 0.00 | 0.52 | 0.79 | 0.27 | 1.6 |
| Libya | 0.00 | 9.68 | 5.16 | 0.16 | 0.32 | 0.16 | 0.97 | 0.81 | 17.3 |

**Table S16.** Cancer Medical devices per 1,000,000 inhabitants in Northern African region.

| Country | MAM | CT | MRI | PET | GC | LA | R | TU | Total |
|---|---|---|---|---|---|---|---|---|---|
| Lesotho | 0.00 | 0.00 | 0.00 | 0.00 | 0.00 | 0.00 | 0.00 | 0.00 | 0.0 |
| Namibia | 2.16 | 4.78 | 0.87 | 0.00 | 0.87 | 0.00 | 0.43 | 0.43 | 9.5 |
| Botswana | 1.11 | 0.99 | 0.49 | 0.00 | 0.00 | 0.00 | 0.00 | 0.00 | 2.6 |
| Swaziland | 1.28 | 2.40 | 0.00 | 0.00 | 0.00 | 0.00 | 0.00 | 0.00 | 3.7 |
| South Africa | 0.54 | 0.97 | 0.23 | 0.06 | 0.53 | 0.40 | 0.57 | 0.17 | 3.5 |

**Table S17.** Cancer Medical devices per 1,000,000 inhabitants in Southern African region.

| Country | MAM | CT | MRI | PET | GC | LA | R | TU | Total |
|---|---|---|---|---|---|---|---|---|---|
| Benin | 0.69 | 0.29 | 0.00 | 0.00 | 0.00 | 0.00 | 0.00 | 0.00 | 1.0 |
| Togo | 0.46 | 0.73 | 0.15 | 0.00 | 0.00 | 0.00 | 0.00 | 0.00 | 1.3 |
| Burkina Faso | 0.53 | 0.65 | 0.06 | 0.00 | 0.06 | 0.00 | 0.00 | 0.00 | 1.3 |
| Cabo Verde | 8.86 | 2.00 | 2.00 | 2.00 | 0.00 | 0.00 | 0.00 | 0.00 | 14.9 |
| Côte d'Ivoire | 0.00 | 0.69 | 0.15 | 0.00 | 0.00 | 0.00 | 0.00 | 0.00 | 0.8 |
| Mauritania | 1.01 | 1.54 | 0.77 | 0.00 | 0.00 | 0.26 | 0.26 | 0.00 | 3.8 |
| Mali | 0.20 | 0.20 | 0.00 | 0.00 | 0.07 | 0.07 | 0.07 | 0.00 | 0.6 |
| Senegal | 0.23 | 0.35 | 0.14 | 0.00 | 0.07 | 0.00 | 0.07 | 0.07 | 0.9 |
| Gambia | 0.59 | 1.08 | 0.54 | 0.00 | 0.00 | 0.00 | 0.00 | 0.00 | 2.2 |
| Ghana | 0.00 | 0.15 | 0.08 | 0.00 | 0.08 | 0.00 | 0.08 | 0.08 | 0.5 |
| Guinea | 0.00 | 0.00 | 0.00 | 0.00 | 0.00 | 0.00 | 0.00 | 0.00 | 0.0 |
| Nigeria | 0.00 | 0.00 | 0.00 | 0.00 | 0.00 | 0.05 | 0.07 | 0.03 | 0.2 |
| Niger | 0.38 | 0.17 | 0.00 | 0.00 | 0.00 | 0.00 | 0.00 | 0.00 | 0.6 |
| Guinea-Bissau | 0.00 | 0.00 | 0.00 | 0.00 | 0.00 | 0.00 | 0.00 | 0.00 | 0.0 |
| Sierra Leone | 0.00 | 0.33 | 0.00 | 0.00 | 0.16 | 0.00 | 0.00 | 0.00 | 0.5 |
| Liberia | 0.00 | 0.00 | 0.00 | 0.00 | 0.00 | 0.00 | 0.00 | 0.00 | 0.0 |

**Table S18.** Cancer Medical devices per 1,000,000 inhabitants in Western African region.

## SUPPLEMENTARY FIGURES

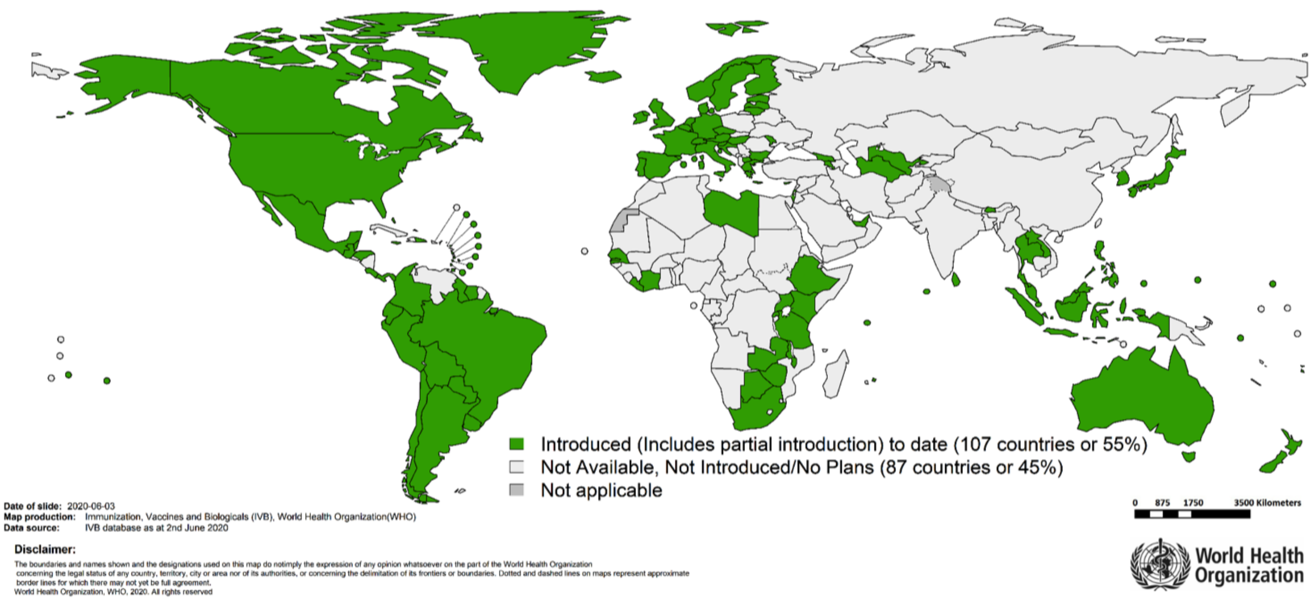


Figure S1: Countries with HPV vaccine in the national immunization program. WHO/ Immunization, Vaccines and Biologicals database, as of 31 March 2017. Available at WHO Vaccine in National Immunization Program Update, June 2020.

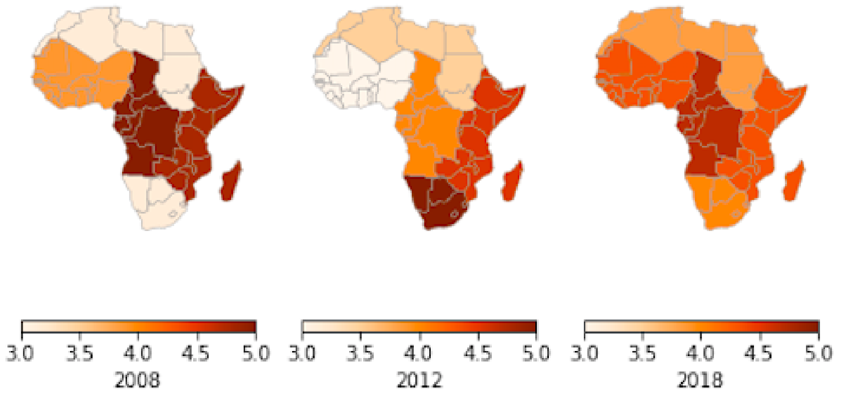


Figure S2: Better resolution of Stomach cancer incidence cases and incidence rates in different African regions per 100,000 inhabitant.

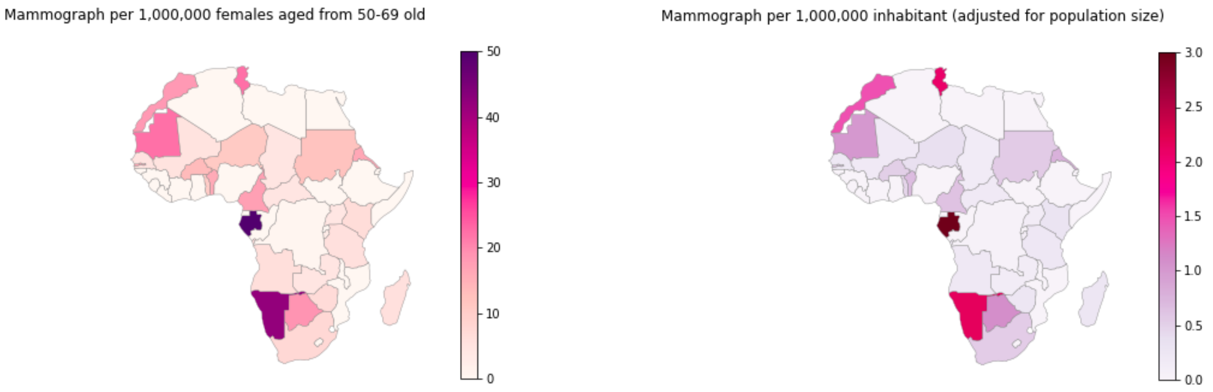


(a) (b)

Figure S3: Densities of Mammographs per 1,000,000 inhabitants were adjusted by multiplying the number of mammographs (available data are the density per 1,000,000 females aged from 50-69 old) by the population percentage of women between 50 and 69 years old; (a) Mammograph density per 1,000,000 females aged from 50-69 old. For Gabon (73.12), Seychelles (127.73) and Cabo Verde (140.60), the mammograph densities are not represented in the map; (b) Mammograph density per 1,000,000 inhabitant. For Mauritius (6.01), Seychelles (11.75) and Cabo Verde (8.86), the mammograph densities are not represented in the map.
